# Cardiovascular Effects of PCB 126 (3,3’,4,4’,5-Pentachlorobiphenyl) in Zebrafish Embryos and Impact of Co-Exposure to Redox Modulating Chemicals

**DOI:** 10.3390/ijms20051065

**Published:** 2019-03-01

**Authors:** Elisabet Teixidó, Marta Barenys, Ester Piqué, Joan M. Llobet, Jesús Gómez-Catalán

**Affiliations:** 1 Department of Bioanalytical Ecotoxicology, Helmholtz Centre for Environmental Research—UFZ, Permoserstraße 15, 04318 Leipzig, Germany; 2 GRET-Toxicology Unit, Department of Pharmacology, Toxicology and Therapeutic Chemistry, Faculty of Pharmacy and Food Sciences, University of Barcelona, 08028 Barcelona, Spain; mbarenys@ub.edu (M.B.); pique.ester@gmail.com (E.P.); jmllobet@ub.edu (J.M.L.); jesusgomez@ub.edu (J.G.-C.)

**Keywords:** cardiovascular toxicity, phenotype, zebrafish embryo, redox modulators, qPCR

## Abstract

The developing cardiovascular system of zebrafish is a sensitive target for many environmental pollutants, including dioxin-like compounds and pesticides. Some polychlorinated biphenyls (PCBs) can compromise the cardiovascular endothelial function by activating oxidative stress-sensitive signaling pathways. Therefore, we exposed zebrafish embryos to PCB126 or to several redox-modulating chemicals to study their ability to modulate the dysmorphogenesis produced by PCB126. PCB126 produced a concentration-dependent induction of pericardial edema and circulatory failure, and a concentration-dependent reduction of cardiac output and body length at 80 hours post fertilization (hpf). Among several modulators tested, the effects of PCB126 could be both positively and negatively modulated by different compounds; co-treatment with α-tocopherol (vitamin E liposoluble) prevented the adverse effects of PCB126 in pericardial edema, whereas co-treatment with sodium nitroprusside (a vasodilator compound) significantly worsened PCB126 effects. Gene expression analysis showed an up-regulation of *cyp1a*, *hsp70*, and *gstp1*, indicative of PCB126 interaction with the aryl hydrocarbon receptor (AhR), while the transcription of antioxidant genes (*sod1*, *sod2*; *cat* and *gpx1a*) was not affected. Further studies are necessary to understand the role of oxidative stress in the developmental toxicity of low concentrations of PCB126 (25 nM). Our results give insights into the use of zebrafish embryos for exploring mechanisms underlying the oxidative potential of environmental pollutants.

## 1. Introduction

Congenital heart defects (CHD) constitute the largest group of congenital anomalies [1]. Although the etiology of the majority of CHD remains unknown, it is likely to be multifactorial, with roles for both genetic and environmental causes. There are several epidemiological studies linking maternal exposure to environmental pollutants with occurrence of a wide range of CHD [2] and experimental studies demonstrate that the developing cardiovascular system is a sensitive target of many environmental pollutants, including dioxins, dioxin-like polychlorinated biphenils (DLCs), and some pesticides [3]. Moreover, accumulating evidence indicates that exposure to environmental chemicals contribute to cardiovascular disease risk, incidence, and severity [4].

Fish are among the most sensitive vertebrates to DLC-induced teratogenicity [5]. Although fish are more sensitive to these effects than mammals are, the developmental effects observed are similar to other vertebrates (mammals and birds) and phenotypically resemble some birth defects in humans [6]. The hallmark endpoints after DLC exposure in fish consists of circulatory failure, edema, craniofacial malformation, and growth retardation leading to lethality [7,8]. 3,3’,4,4’,5-Pentachlorobiphenyl (PCB126) is the most representative coplanar congener of dioxin-like PCBs and has similar structure and biological effects to 2,3,7,8-tetrachlorodibenzo-p-dioxin (TCDD). PCB-mediated dysfunction in the vascular endothelium has been linked to increased oxidative stress mediated through the activation of a cytochrome P450 oxidase, CYP1A, following the activation of the Ah receptor (AhR) [9]. While AhR activation seems to be a prerequisite for DLCs toxicity [10], the identity of the trigger gene or genes regulating teratogenesis remains unknown.

Zebrafish (*Danio rerio*) has received increasing attention as an animal model for understanding chemical effects during development and to study diseases [11]. Particularly, its heart resembles that of a human embryo at three weeks of gestation and effects on the cardiovascular system can be easily assessed in living zebrafish using the microscope [12]. Additionally, unlike mammals, organs and tissues of zebrafish embryo do not depend on the cardiac output for oxygen delivery. Embryos rely on oxygen diffusion through the skin from the swimming medium up to 14 days post-fertilization (dpf) [13], thereby allowing a detailed analysis of animals with severe cardiovascular defects. 

Previously studies have been shown that PCB126 exposure in zebrafish at high concentration (32 to 128 µg/L) produces oxidative stress [14,15]. However, there are contradictory data concerning whether that is true at the low concentrations at which the phenotypic effect is still present [16]. The purpose of the current study was to investigate if oxidative stress is a component of the developmental toxicity of PCB126 in zebrafish at a low concentration level with established phenotypic cardiovascular effects. For that reason, we have first characterized the cardiovascular effects of PCB126 in zebrafish embryos and subsequently evaluated the potential for a set of redox modulator chemicals to reverse the effects of PCB126. 

## 2. Results

### 2.1. Concentration-Dependent Cardiovascular Toxicity of PCB126

**Figure 1:**
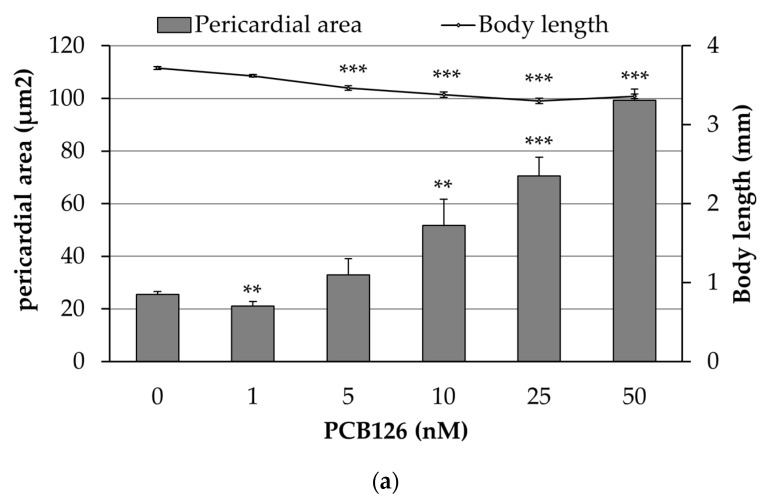

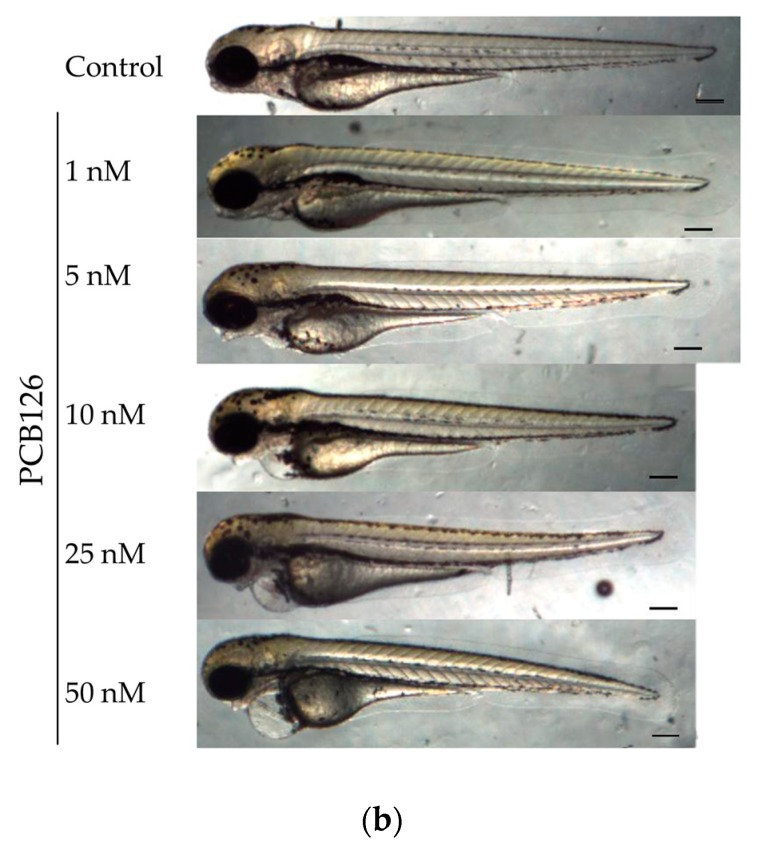
Concentration-dependent morphological effects of PCB126 exposure: (**a**) Mean pericardial sac area and body length of embryos exposed to increased concentration of PCB126. Values are mean ± SEM. Asterisks represent statistical significance at * *p* < 0.05, ** *p* < 0.01, and *** *p* < 0.001; *n* = 4 pools of five embryos (two biological replicates). (**b**) Representative images of embryos at 80 hpf exposed to increasing concentrations of PCB126 (Scale bar = 200 µm).

Embryos were exposed to increasing concentrations of PCB126 (1, 5, 10, 25, and 50 nM) and vehicle (embryo medium with 0.01% acetone) in order to characterize the concentration-dependent cardiovascular toxicity of the compound. As Figure 1 shows, exposure to PCB126 produced a concentration-dependent increase in the pericardial sac area and a reduction on the body length of the larvae with a correlation coefficient of *r* = −0.78. Moreover, the heart of PCB126 exposed embryos (25 nM) failed to loop correctly (observed as a linear heart tube), and at high concentrations (50 nM) embryos also showed ventricular standstill (Supplementary video file 1). 

**Figure 2:**
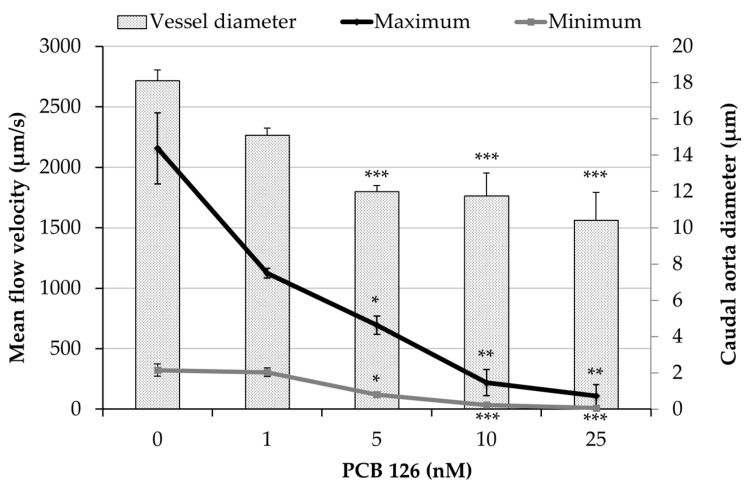
Effects of PCB126 on peripheral blood flow. Maximum and minimum caudal aortic flow velocity after exposure of embryos to PCB126. Vessel diameter plotted on secondary axis. Values are mean ± SEM (*n* = 6 larvae). Asterisks represent statistical significance at * *p* < 0.05, ** *p* < 0.01, and *** *p* < 0.001.

To determine whether PCB126 exposure disrupted peripheral blood circulation, we examined the maximum and minimum caudal aortic blood flow. As Figure 2 shows, exposure to PCB126 produced a concentration dependent decrease in peripheral blood velocity as well as a reduction in the diameter of caudal aortic vessel that resulted in a significantly decreased mean flow from concentration 1nM of PCB126 (Table 1). The reduction on peripheral blood flow circulation also came with a reduction in red blood cells passing through the caudal aortic vessel (Supplementary video file 2). Embryos exposed to PCB126 showed a significantly reduced cardiac output but an unaltered heart rate at 3 dpf (Table 1).

### 2.2. Influence of Redox-Modulators on the Cardiovascular Toxicity Induced by PCB126

Embryos were co-exposed to several redox-modulating chemicals (Table 2) to determine whether a more reduced or oxidized redox level would alter the gross defects observed after PCB126 exposure (pericardial effusion severity or body length). The concentration of PCB126 used was 25 nM, concentration that was found to produce a fully and uniform phenotype in zebrafish using our protocol. Each redox-modulating chemical was first tested independently at increasing concentrations and the maximum tolerable concentration (MTC) was established and subsequently used in the co-exposure experiments. 

We attempted to modulate deformities by decreasing glutathione (GSH) levels with dimethyl maleate (DEM) and increasing GSH pools with N-acetyl cysteine (NAC). Co-treatment with NAC (100 µM) did not prevent deformities and co-treatment with DEM (0.05 µM) failed to worsen deformities (Table 3). We also examined whether several antioxidants could modulate deformities produced by PCB126. Co-treatment with lipoic acid (10 µM) did not ameliorate pericardial effusion and growth of the PCB126 treated larvae at 3 dpf (Table 3), but produced a non-significant increase in pericardial area compared to PCB126. A dual role of lipoic acid as antioxidant/pro-oxidant molecule has already been described and could be related to such a result [17].

**Figure 3:**
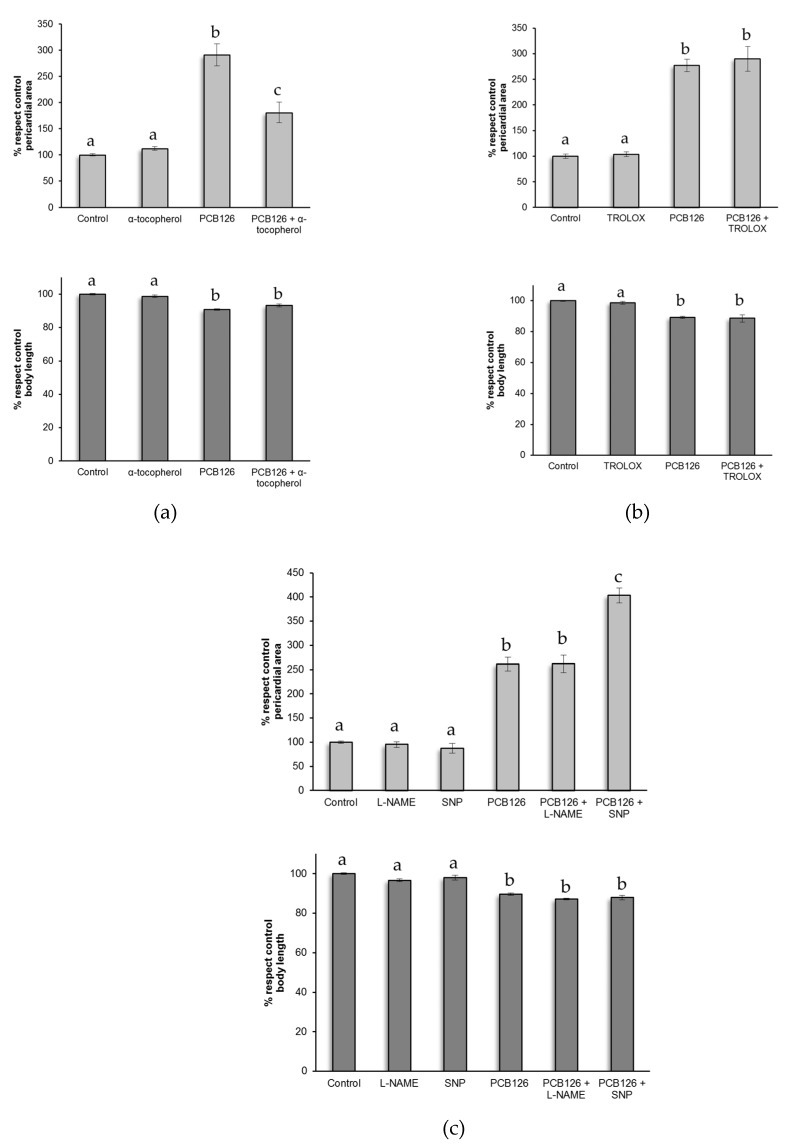
Pericardial sac area and body length of larvae at 80 hpf exposed to PCB126 and co-exposed to (**a**) α-tocopherol (100 µM), (**b**) Trolox (15 µM), (**c**) L-NAME (100 µM), and SNP (250 µM). Data are presented as mean ± SEM on *n* = 6–9 pools of three embryos. Different letters on top of the bars indicate statistically significant differences with *p* < 0.05.

Co-treatment with α-tocopherol (vitamin E liposoluble) at 100 µM ameliorated pericardial sac area, but did not prevent PCB126 induction of pericardial effusion and shortening of body length (Figure 3a). On the other hand, pericardial effusion and shortening of the body length was not prevented by co-treatment with a soluble analogue of vitamin E (Trolox) at 15 µM (Figure 3b). Studies showed that coplanar PCBs have proinflammatory effects on vascular endothelial cells [18] through endothelial nitric oxide synthase (eNOS) signalling. Therefore, we attempted to modulate this effect by co-exposing embryos to indomethacin, a non-steroidal anti-inflammatory compound, and quercetin, a flavonoid with anti-inflammatory properties. Embryos co-exposed either to indomethacin (30 µM) or quercetin (12 µM) still exhibited pericardial effusion and shortening of the body length at 3 dpf compared to control group (Table 4). As shown in Figure 3c, co-exposure to the nitric oxide synthase inhibitor, Nω-Nitro-L-arginine methyl ester hydrochloride (L-NAME, 100 µM), did not result in any significant change in pericardial sac area and body length at 3 dpf. However, co-treatment with a nitric oxide donor, sodium nitroprusside (SNP, 250 µM), worsened the pericardial sac area but did not significantly shorten body length.

### 2.3. Gene Expression Analysis of Oxidative Stress Related Genes 

To determine the redox status of zebrafish embryo exposed to PCB126, gene expression of several antioxidant genes was analyzed by quantitative RT-PCR. Alteration of the redox status was examined in embryos treated with PCB126 at a concentration of 25 nM and after dysmorphogenesis at 3 dpf. The targeted genes involved in the defense against oxidative stress (*gpx1a*, *sod1*, *sod2*, *cat*, and *gstp1*), *cyp1a*, related to the AhR machinery, and *hsp70* involved in the cellular stress response. *Cyp1a* expression increased by nearly 200-fold in embryos exposed to PCB126. We also observed a significant fold change induction of about 1.7 for *hsp70* and *gstp1* genes. For the remaining genes (*gpx1a*, *sod1*, *sod2*, and *cat*), no alteration in expression was observed (Table 4).

## 3. Discussion

The results of this study demonstrate that PCB126 developmental exposure elicits a cardiovascular concentration-dependent toxicity in zebrafish embryos. PCB126 exposure leads to a concentration-dependent increase in pericardial sac area, which is in accordance with previous reports by Jönsson et al. [7], where craniofacial malformations, heart malformations (hypotrophy and reduced looping), slower and weaker heartbeats, impaired circulation, and immobility were also described. Our study also shows that PCB126 exposure leads to a concentration-dependent shortened body length, being the LOAEC 5 nM of PCB126 (Figure 1). This reduction in body length could suggests an impairment of the growth of the larva, but it is also an effect often observed when there is a cardiovascular defect [19], and, in this case, a significant reduction in maximum blood flow at concentrations not affecting body length was detected. An increased pericardial sac area was moderately correlated (*r* = −0.78) with reduced body length. 

Chemicals that are AhR agonists are known to reduce blood flow in trunk vessels of zebrafish embryos at 72 hpf or later [20]. In our study, exposure to PCB126, a known AhR agonist, significantly reduced peripheral blood circulation and aortic caudal vessel diameter in a concentration-dependent way from concentration of 5 nM (Figure 2). Particularly, reduction in blood vessel diameter suggests impairment in endothelial cell function. Considering that, hemodynamic changes play an important role in blood vessel formation and changes in blood flow can lead to severe vascular distortion [21]. Grimes et al. [22] have already strongly suggested a link between hemodynamic forces and myocardial dysmorphogenesis produced by PCB126. In line with this, the observed significant reduction on peripheral blood flow circulation came together with a reduction in red blood cells passing through the caudal aortic vessel (Supplementary video file 2), and cardiac malformation (no heart looping). However, it is not possible to discern whether the PCB126-heart phenotype we observed is secondary to the endocardial disruption and hemodynamic impairment or not. It should be noted that measurement of peripheral blood flow provides more accurate information about the blood pumped by the heart than measuring cardiac output. This is because the method used to measure cardiac output does not take into account a possible blood reflux between chambers. In any case, PCB126 exposure produced a significant decrease in cardiac output from concentration of 5 nM (Table 3). This reduction in cardiac output occurring at the same time was not related to an altered heart rate but to the abnormal morphogenesis of the heart ventricle observed in PCB126 exposed embryos. Antkiewicz et al. [10] also observed a significantly decreased stroke volume and cardiac output, with no effect on heart rate after TCDD treatment. In our case, some treated embryos at high concentrations showed reflux of blood between chambers or a contractile ventricle chamber without blood passing through it (Supplementary video file 2) that made measurement of cardiac output difficult. 

As mentioned before, PCB126 is a strong agonist for AhR, a ligand-activated cytosolic transcription factor that induces the expression of a battery of genes involved in xenobiotic metabolism. The activity of these genes contributes to the formation of reactive oxygen species that can subsequently lead to cellular oxidative stress, lipid peroxidation, and DNA fragmentation [23]. Some studies indicate that an increase in cellular oxidative stress and an imbalance in antioxidant status are critical events in PCB-mediated induction of inflammatory genes and endothelial cell dysfunction [18]. Therefore, in this study we sought to determine the role of redox imbalance in mediating the cardiac toxicity caused by PCB126 in the developing zebrafish. For that reason, embryos were exposed to several redox modulating chemicals to demonstrate if oxidative stress is a component of the developmental toxicity of PCB126. Nine redox modulating chemicals were tested in the zebrafish that are known to modulate different redox mechanisms: increase or decrease in GSH pools (NAC [24] and DEM [25], respectively), free radical scavengers (Lipoic acid [26], vitamin E [14], Trolox [26]), anti-inflammatory agents (indomethacin [27], quercetin [28]), inhibitor of NO synthase (L-NAME [29]), and a nitric oxide donor (SNP [30]). Among all the redox modulating chemicals tested we have observed that PCB126 toxicity could only be modulated by vitamin E. We could observe only a partial amelioration in the pericardial sac area and no significant effect in body length of larvae, in contrast with what was observed in the study of Na et al. [14]. This difference could be due to differences in experimental protocol. On the other hand, our results demonstrated that SNP, a nitric oxide donor, worsened pericardial effusion induced by PCB126 exposure. Pelster et al. [29] demonstrated that the vasodilator effect of NO contributes to changes in local blood flow, and modifications of shear stress. Endothelial cells are able to convert mechanical stimuli into intracellular signals that affect cellular functions (e.g., permeability or remodeling) [31]. Some studies have shown that PCB126 decreases NO production and alters the expression of eNOS in human umbilical vein endothelial cells [32,33] and increases endothelial production of the inflammatory mediator IL-6 [18]. Additionally, it has been suggested that NO can induce COX activity and subsequent production of proinflammatory prostaglandins [34]. Cyclooxygenase-2 (Cox-2) enzymes have been proposed to be involved in some DLC-mediated toxicities [35]. Particularly, PCB126 showed a concentration dependent increase in COX-2 expression [36]. Our results suggest that NO signalling pathway is involved in PCB126 induced endothelial cell permeability dysfunction, although more studies with inhibitors or gene knockdown specific for zebrafish COX-2 are required for further understanding the roles of zebrafish nitric oxide and COX-2 involvement in the cardiotoxicity elicited by PCB126. 

It is well established that PCB126 binds to the AhR and induces the expression of a battery of genes (the AhR gene battery), including *cyp1a*. PCB126 has been shown to markedly contribute to cellular oxidative stress in endothelial cells in vitro by inducing *cyp1a* activity and decreasing vitamin E content [37]. Accordingly in our study, embryos exposed to PCB126 during 24 h showed a strong induction of *cyp1a* at 3 dpf (Table 4). Our results are in line with previous reports [7] demonstrating the largest fold change between basal and induced expression on day 3 (236 ± 34 fold the control). Besides, our analysis shows significant fold induction for HSP70 gene after PCB126 exposure. Heat shock proteins (HSPs) are a highly conserved, ubiquitously expressed family of stress response proteins, which are expressed at low levels under normal physiological conditions. HSPs can function as molecular chaperones, facilitating protein folding, preventing protein aggregation, or targeting improperly folded proteins to specific degradative pathways [38]. Moreover, it has been demonstrated that HSP activity is required for physiological responses, such as endothelial cell migration and blood vessel repair specifically, as *hsp70* morphants of zebrafish have impaired vessel development and sprouting [39]. On the other hand, the expression of genes related to the NRF2 pathway was not altered after exposure to 25 nM of PCB126 at 3 dpf, but there was the upregulation of *gstp1* by PCB126. There are contradictory data concerning the role of NRF2 in regulation of phase II enzymes in zebrafish [16,40,41]. Moreover, some studies have been shown that knockdown of *gstp2* did not serve as a protective function against the cardiac toxicity caused by PCB126 [42]. 

Although the production of PCBs has been strictly regulated in most industrialized countries since the late 1970s, they are still prevalent in the environment due to their high persistence and resistance to bio-degradation. The most abundant PCB congener in serum is usually PCB153, a congener that does not display “dioxin-like” activity. As an approach to compare “dioxin-like” compound concentrations in serum or breast milk with the concentrations used in this study, we have calculated the corresponding toxic equivalent (TEQ). The total TEQ concentrations detected in these biological samples were in the pg/L range, being the highest level in blood around 142.4 pg/L [43]. In contrast, our study is in the range of 326-1630 ng/L. However, how the effect of concentrations in zebrafish embryos can be related to appropriate effect levels in mammalian models is still a major issue for future research. In our study, nominal concentrations were used and may represent a limitation. Under short-term exposures, a steady state may only be reached for test substances with lower hydrophobicity and rarely reached for more hydrophobic substances [44], leading to an underestimation of the apparent short-term toxicity of PCB126. Therefore, the potential health risk for the developing human to “dioxin-like” compounds still needs further investigation.

## 4. Materials and Methods

### 4.1. Zebrafish Maintenance and Egg Production

Adult female and male zebrafish were obtained from a commercial supplier (Pisciber, Barcelona) and housed separately in a closed flow-through system in standardized dilution water (ISO, 1996; 2 mM CaCl_2_·2 H_2_O; 0.5 mM MgSO_4_·7 H_2_O; 0.75 mM NaHCO_3_; 0.07 mM KCl). Fish were maintained at 26 ± 1 °C under a 14:10 light:dark cycle. The study was approved by the Ethic Committee for Animal Experimentation of the University of Barcelona (398/14, Mai 2014) and by the Department of Environment and Housing of the Generalitat de Catalunya with the license number DAAM 9967 (Mai, 2018).

Embryos were collected after natural spawning of adult zebrafish, as previously described [45]. Embryos used in the experiments were held in 0.3× Danieau’s solution (5.8 mM NaCl; 0.23 mM KCl; 0.13 mM MgSO_4_·7 H_2_O; 0.2 mM Ca(NO_3_)2; 5 mM HEPES; pH 7.4) at 27 °C and at 14:10 light:dark cycle. All exposures were performed in glass crystallization dishes. 

### 4.2. Exposure to PCB126 

Groups of 25 embryos at approximately 8 h post-fertilization (hpf) were exposed to increasing concentrations of PCB126 (Dr. Ehrenstorfer GmbH, Augsburg, Germany) or carrier solvent (0.01% acetone, *v*/*v*) in 20 mL of 0.3× Danieau’s solution. After 24 h, the exposure test solution was removed and replaced with fresh 0.3× Danieau’s solution. Embryos were held, with daily changes of the medium, until 3 dpf. 

### 4.3. Exposure to Redox Modulating Chemicals

Embryos were exposed to chemical modulators at 4 hpf and during the whole test until 3 dpf, renewal of medium was done every 24 h. All redox modulating chemicals were dissolved in 0.3× Danieau’s solution, except lipoic acid, α-tocopherol, and indomethacin that were initially prepared in 100% DMSO and subsequently diluted in embryo medium (0.05% DMSO, *v*/*v*). Trolox stock solution was prepared at 2 mM in 0.3× Danieau’s solution and filtered (0.22-μm syringe filter) (GE Healthcare Whatman®, Chicago, Illinois, USA). The concentration was adjusted using absorption at 290 nm and extinction coefficient of 2350/M/cm. A preliminary range finding test was performed in order to determine the maximum tolerable concentration (Table 2), defined as the maximum concentration tested that does not produce a significant increase in mortality or dysmorphogenesis. At 3 dpf, induction of the pericardial sac area and body shortening was compared between embryos treated with PCB126 alone and embryos co-treated with PCB126 chemical modulator.

### 4.4. Heart Rate

Cardiovascular function of embryos was evaluated by time-lapse recording using a high-speed digital camera (Exilim, CASIO). Sequential images of the heart (480 frames per second –fps–) were obtained with the embryo positioned on its side from the lateral position and with a duration of 5 s. To avoid the movement of zebrafish embryo during recoding, embryos were anaesthetized by adding tricaine to embryo medium and mounted in 3% methylcellulose on double depression slides. The final concentration of tricaine was 0.004% (*w*/*v*), which has shown to not affect heart rate in zebrafish embryos [46].

To quantify heartbeat in the zebrafish, video recording were analyzed with ImageJ [47]. A plot of dynamic pixels was obtained by selecting an area of the heart ventricle with high deviation of pixel intensity by means of the z projection function. The frequency as frames/beat was obtained by analyzing the waveform of dynamic pixels by Short-term Fourier Transform using Statgraphics software (Statpoint Technologies Inc., Virginia, Washington D.C., USA). Then, the heart beat frequency (beats/min) was calculated taking into account the speed of video recording (480 fps). 

### 4.5. Stroke Volume and Cardiac Output

To ensure that a comparable plane of focus was examined for ventricular analysis, zebrafish hearts were imaged in a standardized lateral right position, with the ventricle clearly visible in the plane of focus throughout the cardiac cycle. The atrium usually lay outside the plane of focus. Ventricular performance of embryos was analyzed by identifying frames that captured ventricular end systole and end diastole in the sequential still frames (as shown in [48]). From the ventricle, image major and minor axes measurements were extracted and exported to an Excel spreadsheet. Calculation of ventricle volume during systole and diastole was based on the formula for the volume of a prolate spheroid (V = 4πab2/3), where “a” represents the major axe radius and “b” the minor axe radius of the ventricle image. The stroke volume (SV), the volume of blood ejected by the ventricle in one heart beat was calculated using the Equation (1).

SV (nl) = (end-diastolic volume – end-systolic volume)(1)

Cardiac output (CO), the volume of blood ejected by the heart in one minute, was calculated using Equation (2).

CO (nl/min) = SV (nl) × heart rate (beats/min)(2)

### 4.6. Peripheral Blood Flow

To identify a reproducible location for analysis of blood circulation, the dorsal aorta adjacent to the cloaca was chosen as a defined imaging landmark. Video recordings of the dorsal aorta at 240 fps were processed with ImageJ. A scan line was placed parallel on the blood flux on the stack of images, and a series of lines were obtained whose slopes were inversely proportional to cellular velocity. OrientationJ plugin [49] of ImageJ was used in order to calculate cellular velocity from the scanned lines. 

### 4.7. Pericardial Sac Area and Body Length

To quantify the degree of increase in the pericardial sac area and body shortening, lateral images were obtained for at least 10 larvae mounted in 3% methylcellulose. By tracing the boundaries of the pericardial space with ImageJ software, the pixel area was obtained. For body length, a line starting at the anterior-most point of the head and ending at the tail end was measured.

### 4.8. Quantitative RT-PCR

Total RNA was extracted from a pool of 15 embryos per sample homogenized zebrafish larvae (3 dpf) using TRIZOL® reagent (Life Technologies S.A., Carlsbad, California, USA) according to the manufacturer’s instructions. RNA quantity and quality was analyzed spectrophotometrically using a NanoDrop ND1000 (NanoDrop Technologies, Wilmington, Delaware, USA). RNA samples were stored at −80 °C until use. cDNA was produced from 2 µg of total DNAse treated RNA samples using RevertAid™ Reverse Transcriptase (MBI Fermentas, Thermo Fisher Scientific, Inc., Waltham, Massachusetts, USA) in 20 µL. 

Real-time PCR reactions were carried out using the SensiMix SYBR Hi-ROX One-Step Kit (Bioline Reagents Ltd., London, UK) and following manufacturer’s instructions. Amplifications were performed in 384-well plates on an ABI 7900HT (AppliedBiosystems, Life Technologies S.A., Carlsbad, California, USA) real-time PCR machine at the Scientific and Technologic Center of UB (CCITUB) under the following thermal cycle: initial denaturation for 10 min at 95 °C, 40 cycles of denaturation for 10 s at 95 °C, annealing for 20 s at 55 °C, and elongation for 20 s at 72 °C. A final denaturation was performed for 30 s at 95 °C. This was followed by generation of a melting curve, starting from 60 °C to 95 °C. Temperature was raised in 0.5 °C increments, holding each temperature for 7 s. Primer sequences were obtained from literature or designed using Primer3 program [50]. Primer sequences and accession numbers of all investigated genes are listed in Supplementary Table S1. *Gapdh* gene was used as a housekeeping gene for qPCR normalization. 

### 4.9. Statistics

Statistical analysis was performed with SPSS 15.0 (IBM, Chicago, USA). One-way analysis of variance (ANOVA) followed by post hoc multi-comparison with Bonferroni’s test was used to analyze homogeneous data of the continuous variables. Significance threshold was established at *p* < 0.05.

The comparative Ct method [51] was used to determine average fold induction of mRNA by comparing the Ct of the target gene to that of the reference gen (*Gapdh*). The fold change obtained for each biological replicate pool of 15 embryos was averaged for treatments. Statistical analysis was performed with REST© software [52].

## 5. Conclusions

PCB126 exposure leads to a cardiovascular failure manifested by the development of pericardial effusion, body shortening, altered peripheral blood flow, and cardiac performance in zebrafish larvae. It is likely that PCB126 disrupts endothelial cell permeability and it seems that altered hemodynamic forces could be implicated. However, the precise nature of this cellular dysfunction and how it leads to abnormal heart morphology remains to be elucidated. The results further suggest that redox modulation did not serve as a protective function against cardiovascular effects caused by PCB126 in developing zebrafish. Oxidative stress may not be the primary factor contributing to the deformities caused by low concentration of PCB126.

## Figures and Tables

**Table 1:** PCB126 exposure decreased peripheral mean blood flow velocity from 1 nM, caused no change in heart rate but significantly decreased cardiac output. The highest concentration tested (50 nM) could not be measured due to the high reduction of blood cells. Values are mean ± SEM on *n* = 6 larvae. Asterisks represent statistical significance at * *p* < 0.05, ** *p* < 0.01, and *** *p* < 0.001.

Treatment	Maximum Blood Flow (nL/min)	Minimum Blood Flow (nL/min)	Heart Rate (beats/min)	Cardiac Output (nL/min)
Control	34.7 ± 6.8	5.2 ± 1.1	215.7 ± 3.4	47.0 ± 6.1
PCB 126 1nM	12.1 ± 0.7 *	3.2 ± 0.3 *	219.4 ± 5.7	35.1 ± 4.4
PCB 126 5 nM	4.7 ± 0.6 *	0.8 ± 0.1 *	215.0 ± 10.7	22.8 ± 1.4 **
PCB 126 10 nM	2.7 ± 1.6 *	0.4 ± 0.3 *	226.8 ± 17.0	14.1 ± 4.7 ***
PCB 126 25 nM	1.7 ± 1.6 *	0.1 ± 0.1 *	209.2 ± 5.8	22.0 ± 5.9 **

**Table 2:** Overview of the chemicals used as modulators in the cardiac toxicity produced by PCB126.

Substance	CAS Number	MTC ^1^ (µM)	Description
N-Acetyl-L-cysteine (NAC)	616-91-1	100	Glutathione precursor
Diethyl maleat (DEM)	141-05-9	0.05	Glutathione depletor
(±)-α-Lipoic acid	1077-28-7	10	Antioxidant and free radical scavenger
(±)-α-Tocopherol	10191-41-0	100	Antioxidant and peroxyl radical scavenger
(±)-6-Hydroxy-2,5,7,8-tetramethylchromane-2-carboxylic acid (TROLOX)	53188-07-1	15	Water-soluble analogue of alpha-tocopherol
Quercetin	117-35-9	12	Flavonoid (mitochondrial ATPase and phosphodiesterase inhibitor)
N_ω_-Nitro-L-arginine methyl ester hydrochloride (L-NAME)	51298-62-5	100	Inhibitor of nitric oxide synthase
Sodium nitroprusside dihydrate (SNP)	13755-38-9	250	Nitric oxide donor
Indomethacin	53-86-1	30	Non-steroidal anti-inflammatory compound

Note: ^1^ Maximum tolerable concentration.

**Table 3:** Pericardial sac area and body length of larvae at 3 dpf exposed to PCB126 and co-exposed to several redox-modulating chemicals. Data are presented as mean ± SEM on *n* = 3–5 pools of three embryos. Different letters on top of the bars indicate statistically significant differences with *p* < 0.05.

Treatment	Pericardial Sac Area *(% Control Vehicle area)*	Body Length *(% Control Vehicle Length)*
Vehicle control	100 ± 4.1 ^a^	100 ± 1.1 ^a^
DEM 50nM	128.7 ± 3.9 ^a^	98.4 ± 0.5 ^a^
PCB126 25 nM	329.5 ± 20.6 ^b^	94.1 ± 0.9 ^b^
PCB126 25 nM + DEM 50 nM	302.5 ± 10.8 ^b^	93.7 ± 0.4 ^b^
Vehicle control	100 ± 2.9 ^a^	100 ± 1.2 ^a^
NAC 100 µM	88.3 ± 8.2 ^a^	101.2 ± 1.4 ^a^
PCB126 25 nM	205.0 ± 21.5 ^b^	94.1 ± 0.5 ^b^
PCB126 25 nM + NAC 100 µM	216.5 ± 24.4 ^b^	92.8 ± 0.9 ^b^
Vehicle control	100 ± 4.9 ^a^	100 ± 0.8 ^a^
Lipoic acid 10 µM	102.9 ± 2.9 ^a^	98.4 ± 1.0 ^a^
PCB126 25 nM	271.6 ± 10.4 ^b^	93.9 ± 0.1 ^b^
PCB126 25 nM + lipoic acid 10 µM	318.9 ± 39.4 ^b^	88.9 ± 0.8 ^b^
Vehicle control	100 ± 3.1 ^a^	100 ± 0.1 ^a^
Quercetin 12 µM	118 ± 6.4 ^a^	98.5 ± 0.6 ^a^
PCB126 25 nM	350.5 ± 13.0 ^b^	91.5 ± 0.5 ^b^
PCB126 25 nM + quercetin 12 µM	340.3 ± 26.4 ^b^	91.6 ± 1.0 ^b^
Vehicle control	100 ± 10.1 ^a^	100 ± 0.3 ^a^
Indomethacin 30 µM	114.9 ± 1.7 ^a^	96.5 ± 0.7 ^a^
PCB126 25 nM	223.9 ± 19.2 ^b^	92.3 ± 0.5 ^b^
PCB126 25 nM + indomethacin 30 µM	246.9 ± 22.8 ^b^	89.6 ± 1.3 ^b^

**Table 4:** Expression of genes involved in the redox status of the embryo after exposure to PCB126 (25 nM) and after deformity development at 3 dpf. Relative expression was calculated respect the housekeeping gene (*gapdh*) and control (0.01% acetone). Data are represented as mean ± SEM of *n* = 3 pools of 15 embryos from different biological replicates. Asterisk indicates significant induction compared to the vehicle control (*p* < 0.05).

Gene	Fold Change
***cyp1a***	199.6 ± 62.1 *
***hsp70***	1.72 ± 0.2 *
***gpx1a***	1.21 ± 0.2
***sod1***	1.03 ± 0.07
***sod2***	1.02 ± 0.06
***cat***	0.91 ± 0.09
***gstp1***	1.74 ± 0.5 *

## Supplementary Materials

Supplementary materials can be found at https://www.mdpi.com/1422-0067/20/5/1065/s1.

## Abbreviations

CHD	Congenital heart defects
DLC	Dioxin-like polychlorinated biphenil
TCDD	2,3,7,8-tetrachlorodibenzo-p-dioxin
PCB126	3,3’,4,4’,5-Pentachlorobiphenyl
AhR	Aryl hydrocarbon receptor
MTC	Maximum tolerable concentration
GSH	Glutathione
DEM	Dimethyl maleate
NAC	N-Acetyl-L-cysteine
TROLOX	(±)-6-Hydroxy-2,5,7,8-tetramethylchromane-2-carboxylic acid
L-NAME	Nω-Nitro-L-arginine methyl ester hydrochloride
SNP	Sodium nitroprusside dihydrate
