# Supplementary material for: Cardiovascular Effects of PCB 126 (3,3’,4,4’,5-Pentachlorobiphenyl) in Zebrafish Embryos and Impact of Co-Exposure to Redox Modulating Chemicals
Source: Int J Mol Sci. 2019 Mar 1;20(5):1065. doi: 10.3390/ijms20051065 (PMC6429282; doi:10.3390/ijms20051065)
Supplement: Supplementary file 1 [file ijms-20-01065-s001.zip › Supplementary material.pdf]

Cardiovascular effects of PCB 126 (3,3',4,4',5-Pentachlorobiphenyl) in zebrafish embryos and impact of co-exposure to redox modulating chemicals

Elisabet Teixidó [1,2,*], Marta Barenys [2], Ester Piqué [2], Joan M. Llobet and Jesús Gómez-Catalán [2]

[1] Department of Bioanalytical Ecotoxicology, Helmholtz Centre for Environmental Research - UFZ, Permoserstraße 15, 04318 Leipzig, Germany 1; elisabet.teixido@ufz.de

[2] GRET-Toxicology Unit, Department of Pharmacology, Toxicology and Therapeutic Chemistry, Faculty of Pharmacy and Food Sciences, University of Barcelona, 08028, Barcelona, Spain.

* Correspondence: elisabet.teixido@ufz.de; Tel.: ++49 341 235 1509

Supplementary material

Table S1. Target genes and specific primers used in this study.

Gene name	Common name	Accession number	Primer sequence (Forward and reverse 5′-3′)	Reference
GAPDH	*Glyceraldehyde-3-phosphate dehydrogenase*	NM_001115114	AGGCAGAAGGCGGCAAAC<br>AAGACACCAGTAGACTCCACAAC	[1]
HSP70	*Heat shock cognate 70-kd*	NM_131397	GGTTCGATGACCCTGTAGTG<br>GGACCATGGAGGAAATCTCT	This study
GPX1A	*Gluthatione peroxidase 1a*	NM_001007281	ACCTGTCCGCGAAACTATTG<br>TGACTGTTGTGCCTCAAAGC	This study
CAT	*Catalase*	NM_130912	GCTCCACCACGTGAATAAAC<br>GGAGCTCAACTCTTCATCCA	This study
SOD1	*Superoxide dismutase 1, soluble*	Y12236	TGTCAGCGGGCTAGTGCTT<br>GTCGTCTGGCTTGTGGAGTG	[2]
SOD2	*Superoxide dismutase 2, mithocondrial*	NM_199976	AGCCAAAGTCACGCTTTATG<br>GGTGATGTGACAACCCAAGT	This study
CYP1A	*Cytocrome P450, family 1, subfamily A*	AF210727	CGACTGCAAAGAGAATTGGA<br>CTTTGGATGTGCAGTGAGGA	[3]
GSTP1	*Glutathione S-transferase pi 1*	NM_131734.3	TCATTGACGTGATGAACGAC<br>ATCTGATCACCAACCAGGAA	This study

[1] Klüver, N.; Yang, L.; Busch, W.; Scheffler, K.; Renner, P.; Strähle, U.; Scholz, S. Transcriptional Response of Zebrafish Embryos Exposed to Neurotoxic Compounds Reveals a Muscle Activity Dependent hspb11 Expression *PLoS One* **2011,** 6, e29063.

[2] Banni, M.; Chouchene, L.; Said, K.; Kerkeni, A.; Messaoudi, I. Mechanisms underlying the protective effect of zinc and selenium against cadmium-induced oxidative stress in zebrafish Danio rerio. *BioMetals* **2011**, 24, 981–992.

[3] Grimes, A. C.; Erwin, K. N.; Stadt, H. A.; Hunter, G.L.; Gefroh, H. A.; Tsai, H. J.; Kirby, M. L. PCB126 exposure disrupts zebrafish ventricular and branchial but not early neural crest development. *Toxicol Sci* **2008,** 106, 193–205.
